# Efficacy and Safety of Melatonin as an Adjunctive Therapy on Clinical, Biochemical, and Quality of Life in Patients with Ulcerative Colitis 

**DOI:** 10.22037/ijpr.2020.113822.14508

**Published:** 2021

**Authors:** Shabnam Shahrokh, Roja Qobadighadikolaei, Mohammad Abbasinazari, Mehrdad Haghazali, Hamid Asadzadeh Aghdaei, Saeed Abdi, Hedieh Balaii, Neda Khanzadeh-Moghaddam, Mohammad Reza Zali

**Affiliations:** a *Gastroenterology and Liver Diseases Research Center, Research Institute for Gastroenterology and Liver Diseases, Shahid Beheshti University of Medical Sciences, Tehran, Iran. *; b *Department of Clinical Pharmacy, School of Pharmacy, Shahid Beheshti University of Medical Sciences, Tehran, Iran.*; c *Shaheed Rajaei Cardiovascular Medical and Research Center, Tehran, Iran.*

**Keywords:** Melatonin, Ulcerative colitis, Fecal calprotectin, Quality of life, Dietary supplement

## Abstract

Ulcerative colitis (UC) is characterized by recurring episodes of inflammation limited to the mucosal layer of the colon. The exact etiology of UC is unknown, but the role of autoimmunity and activated inflammatory cascade is quite clear. Melatonin possesses anti-inflammatory and immune-modulative properties in animal and clinical trials. The aim of the present study was to evaluate the efficacy and safety of oral melatonin as an adjudicative therapy in clinical, biochemical, and quality of life in UC patients. Thirty patients diagnosed with mild to moderate UC, were randomly allocated to either receive melatonin (3 mg/d) or the placebo group for three months. Simple clinical colitis activity index (SCCAI), fecal calprotectin (FC), C-reactive protein (CRP), Erythrocyte Sedimentation Rate (ESR), and Sf-36 questionnaire have been used for assessment at the baseline and the end of the trial. Melatonin significantly improve SCCAI score, FC, role-emotional, energy and general health relative to placebo (*p* = 0.03, 0.05, 0.002, 0.032, 0.004 respectively). Regarding CRP, ESR, and the other components of SF-36 there is not any significant difference between melatonin and placebo group. Melatonin supplementation over a three-month period is effective and safe in improving clinical index, FC, and some quality of life in patients with mild to moderate UC.

## Introduction

Ulcerative colitis (UC) is a subtype of inflammatory bowel disease (IBD). It is a chronic disease that can involve any part of the colon, starting with mucosal inflammation in the rectum and extending proximally in a continuous fashion (1). The etiology of UC has not been perfectly known but multiple factors such as genetic, oxidative stress and environmental factors which leads to immune dysregulation have been mentioned (2). There are different medications to achieve clinical remission , such as 5-aminosalicylates, corticosteroids, thiopurines, calcineurin inhibitors, anti-tumor necrosis factor (TNF) agents and anti-adhesion molecules (3). The choice of which drug to use is determined by the presenting disease severity and the location of the disease (4). More than 15% of the UC patients may need to surgery because of they fail to respond to pharmacotherapy interventions (1). The aim of management in UC is first to induce a clinical remission and then induce steroid-free maintenance of remission as much as possible (4). Remission is defined as normalization of bowel movements and cessation of bleeding. Refractory UC could be defined as patients with endoscopically documented active colitis, who fail oral corticosteroids combined with oral and rectal 5- aminosalicylates therapy (5, 6). This states the need for research on new medications, which could be a helpful adjuvant treatment in this chronic disease. For the same reason, the antioxidant vitamins were prescribed in the treatment of UC (7) .

Melatonin (N-acetyl-5-methoxytryptamine) is a hormone first discovered in 1958 in bovine pineal (8). Previous studies demonstrated that multiple tissues such as the gastrointestinal (GI) tract are sites of production and the amount of melatonin is estimated to be 400 to 500 times more than in the pineal gland (9). It has been suggested that melatonin released in the GI tract may play an important role in the GI tract physiology and pathophysiology. In the intestine, melatonin affects physiological consequences such as regeneration of the epithelium and adjustment of its function, modulation of the immune response and decrease in the tone of GI muscles through particular membrane receptors such as melatonin-1 receptor (MT-1), MT-2, and may be through MT-3 (10). Besides melatonin is a potent antioxidant, anti-inflammatory, and antigenotoxic effect by mechanism such as preventing the production of nitric oxide, reducing the level of cytokines, suppressing the enhanced myeloperoxidase activity, and inhibiting the activity of nuclear factor-kappa beta (NF-kb) (9, 10).

The aim of the present study was to evaluate the effect of melatonin as adjunctive therapy on clinical, biochemical parameters, and quality of life in patients with the diagnosis of mild to moderate UC.

## Experimental

The present study is a double-blind randomized clinical trial for the assessment of melatonin 3 mg/d as an adjunctive therapy of UC patients with mild to moderate index. Eligible patients have been selected from two well –known centers of gastrointestinal and liver diseases in Tehran, Iran. 

*Ethics approval and consent to participate*

The protocol of the trial was evaluated and approved by the ethical committee of Shahid Beheshti University of medical sciences with number IR.SBMU.PHARMACY.REC.1398.158. The study has also been registered in Iranian Clinical Trial Registry (IRCT) with number IRCT20121021011192N9.

*Entry criteria*

Inclusion criteria were any patient with age over 20 years old with a confirmed diagnosis of UC with colonoscopy and histology who had simple colitis activity index (SCCAI) score between 3-11 (mild to moderate). Diagnosis of UC was confirmed pathologically by endoscopic data. In addition, the informed consent was obtained from all selected patients before enrolment.

Exclusion criteria were patients with a history of seizure, depression, hepatic impairment (child-pugh B or C), and renal failure. Moreover, the patients who disagreed with the enrolment of the study, IV drug abuser, pregnant or lactating women, mechanical obstruction, perforation of gut, current users of nifedipine or fluvoxamine (due to significant drug interaction with melatonin), and the patients who used any Non-Steroidal Anti-Inflammatory Drugs (due to interfering with results of fecal calprotectin (FC) test) were excluded from the study too. 

*Intervention and follow-up*

Eligible patients were assigned randomization numbers and treated according to protocol study. An online statistical computing web program was used for random assignment. (http://www.graphpad.com/quickcalcs/randomize1.cfm).

Eligible patients were enrolled and randomized in a 1:1 ratio to receive a melatonin tablet (3 mg/d) or a placebo tablet. Melatonin tablets for this trial have been obtained from Nature Made, USA, and placebo tablets have been prepared by pharmaceutical laboratory of Shahid Beheshti University of medical science. When enrolling an eligible patient to the trial, a qualified pharmacist delivered one tablet box labelled with patient codes, depending on their allocation. The pharmacist did not have any role in selecting, evaluating or assessing the participants during the study course. Participants were asked to use either melatonin tablet or placebo for three months. 

At the beginning of trail and after 3 months, clinical activity, sensitive serum biomarkers for active inflammation of the colon (including CRP and ESR), FC, and quality of life were evaluated. 

Assessment of clinical activity was done by using a simple clinical colitis activity index questionnaire (SCCAI-Q). SCCAI-Q, which is appropriate for assessment of patients with UC, is a clinical activity indicator and contain 6 questions: bowel frequency per day, bowel frequency per night, the urgency of defecation, blood in stool, general well-being, and extra-colonic manifestations, scaled from zero to 19 (0-2 remission phase, 3-5 mild, 6-11 moderate, 12-19 severe) (11). Quality of life was determined by using SF36 questionnaire in the participants. The SF-36 is one of the most used generic health-related quality of life questionnaires in medicine; it evaluates the functional condition, the general perception of health, and well-being with high validity and reliability. The SF-36 consists of a multi-item scale of 36 questions that can be translated into eight sections (12). The demographic data, scores of SCCAI and SF36 were recorded before and after three months in all participants. If any serious adverse events occurred during the intervention regarding melatonin or placebo usage, the intervention was withheld, and the patient was excluded from the study. 

*Statistical analysis*

Data were analyzed by using SPSS (Version 21.0; SPSS Inc., Chicago, IL, USA) software. P value equal or less than 0.05 was considered significant. Comparisons between melatonin and placebo groups were performed using independent samples *t*-test, Mann–Whitney test, and Pearson’s Chi-squared test as appropriate. The sample size was calculated due to primary outcome, *i.e*., the changes in the FC levels at 3 months compared with baseline. Fifty points of FC from the baseline value in at least 70% of patients in the melatonin group and 40% of patients in the placebo group was used for sample size calculations (13). To discover a significant difference between the two groups (0.05 significance level, two-sided Fisher’s exact test) with 80% power, the minimum number of patients to be enrolled is estimated at 30 in a 1:1 ratio.

## Results

**Figure 1:**
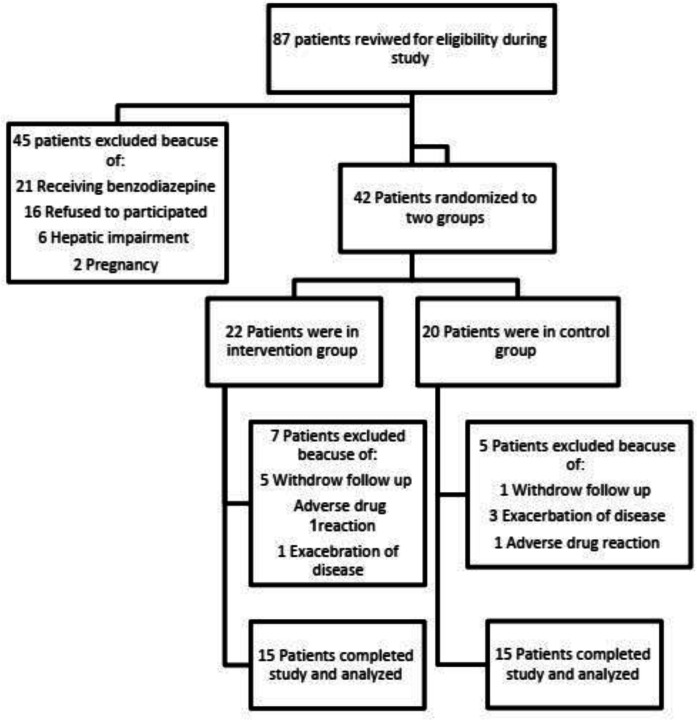
Consort chart of study

Between December 2019 and May 2020, a total of 87 patients with the diagnosis of UC have been screened in mentioned clinics. Fifty-seven of them were excluded and finally 30 patients completed the study. The ﬂowchart of participants throughout the study is shown in Figure 1.

Demographic data and biochemical parameters of participants at the baseline are shown in Table 1. No significant difference was noted regarding age, sex, Body mass index (BMI), duration of UC, and biochemical markers between the groups. 

**Figure 2:**
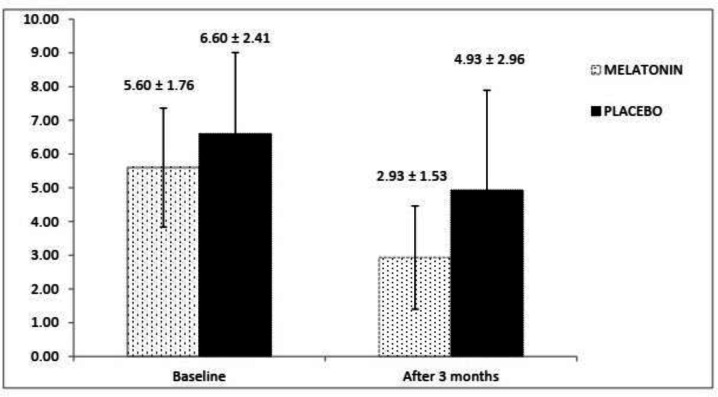
The changes in the SCCAI value in two groups over time

The SCCAI scores at the baseline and end of the study in both groups have been shown in Figure 2. There is not any significant difference regarding SCCAI scores at the baseline between melatonin and placebo groups (*p* = 0.126). Higher SCCAI score were observed in the placebo group compared with melatonin group after 3 months and the difference was statistically significant (*p* = 0.030).

Table 2 shows the descriptive values for the SF-36 in various parts, including physical functioning, role-physical, general health, energy, social functioning, role-emotional, health change, and bodily pain at the baseline and at the end of the study. At the beginning of the trial, there is not any significant difference between the two groups regarding various parts of SF-36; however, there was a significant improvement in the melatonin group compared with the placebo group in role-emotional, energy, and general health at the end of study (*p* = 0.050, *p* = 0.002 and *p* = 0.037 respectively).

In Table 3, biochemical parameters of melatonin and placebo groups have been shown at the end of the study. Statistical analysis showed that the level of ESR and CRP did not change significantly between the two groups at the end of the study (*p *= 0.697, *p* = 0.112 and respectively).

**Figure 3:**
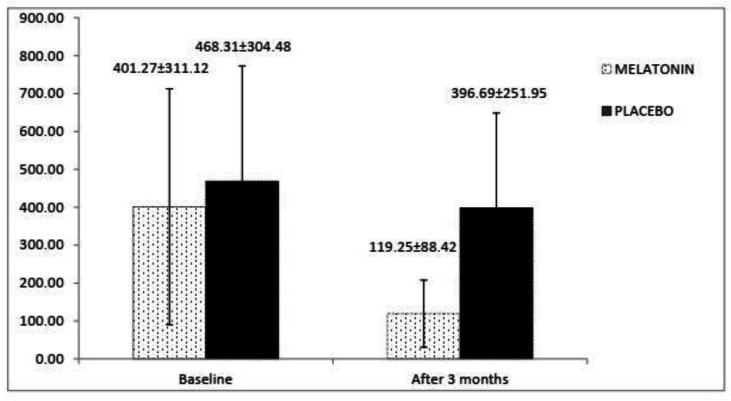
The changes in the Fecal calprotectin level in two groups over time

In Figure 3, amounts of FC have been shown at the begging and the end of the trial. It was determined that baseline FC was not different between the melatonin and placebo groups (*p* = 0.924). At the end of the study, FC level decreased in the melatonin arm (from 401.27 ± 311.12 to 119.25 ± 88.42) versus the placebo arm (from 468.31 ± 304.48 to 396.69 ± 251.95), and analysis showed that the declining trend in the melatonin group compare to the placebo group was significant (*p *= 0.004). 

In general, both melatonin and placebo were tolerated well, but three patients in the melatonin group experienced nightmares during the study. In two cases, this adverse reaction was not severe, and only one of them prefers to exclude the study. One patient in the placebo group has also been excluded because of the occurrence of rash after starting the placebo.

## Discussion

Previous studies have been indicated that melatonin may be act via multiple functions such as reproduction, enhancement of immunomodulation, antioxidant defense, and anti-inflammatory properties. The anti-inflammatory activity of melatonin could be helpful in the treatment of chronic inflammatory diseases such as UC (8). The site of inflammation in this disease is mucosa and submucosa of the colon wall, and mainstay of treatment in mild to moderate UC is mesalamine compounds which modulate local chemical mediators of the inflammatory response and is also postulated to be a local free radical scavenger or inhibitor of tumor necrosis factor in the intestinal wall. Of course, they must bypass the stomach and small intestine and reach the colon finally (14, 15). Although oral melatonin absorbs in the GI tract, absorption in the stomach is the least and the most significant absorption in the rectum and ileum, respectively (16). So it’s logical to administer oral melatonin in the treatment of UC.

Several animal trials have been conducted regarding the effect of oral and rectal administration of melatonin on the suppression of UC, but to the best of our knowledge, there is only one randomized, double-blind clinical trial that evaluated the effects of melatonin 5mg/d as adjuvant treatment of UC which have done by Chojnacki *et al. *(7). In Chojnacki *et al.* study, only assessment of clinical response, anxiety degree, and CRP have been evaluated as the main outcome. We have evaluated the effect of oral melatonin as an adjunctive therapy of UC patients too, but we have considered three different components, including clinical marker (SSCAI), biochemical markers (FC, CRP, and ESR), and quality of life (using SF-36). At the baseline there is not any significant difference between the melatonin and placebo groups regarding any of the above parameters (clinical, biochemical, and quality of life). In other words it seems that both groups are matched, and there is not any confounding factor.

At the end of the study, the difference in clinical activity index in the two groups was one of the most interesting findings of our trail where by using SSCAI, melatonin group had a better index than the placebo group (*p *= 0.030). Chojnacki *et al.* have been evaluated clinical status of UC patients by using Mayo Clinical Disease Activity Index (MCDAI). They have reported that melatonin group had a better index compared with placebo group (*p* = 0.02) (7). Although several indices for UC activity have been developed, there is not any gold standard amongst them (17). There is no acknowledged metric for comparing indices, which vary in their scale of scores and the data collected. Agreement between disease activity categories assigned for each index and the clinical category assigned by a physician with access to all data beyond that recorded by any individual index, were 61% and 67% for the SCCAI and MCDAI respectively. In addition SCCAI does not need physician estimation, laboratory tests or invasive test against the MCDAI. Since SCCAI compared with MCDAI is a purely clinical index for UC patients (18), we prefer to use SCCAI as a clinical marker for assessment in our trial. Concern to our trial and Chojancki *et al.* study, melatonin could be recommended as an adjunctive therapy for optimizing a better clinical index in UC patients.

The recent guideline of the American College of Gastroenterology (ACG) has mentioned that FC can be used in patients with UC as a noninvasive biomarker of disease activity and to assess response to treatment and relapse (18). In previous trial regarding effect of melatonin in UC patients, FC has not been assayed (7) and our trial is the first one to compare effect of melatonin versus placebo on FC level in UC patients. Our results showed that FC level decreased significantly in melatonin group compared with placebo group (*p* = 0.004).

In the current study, ESR and CRP did not significantly differ between the two groups (*p* = 0.697 and *p* = 0.112, respectively). Although the result of Chojancki et al trial demonstrated declined CRP in melatonin group versus placebo group significantly (7), it is important that unlike the FC, both ESR and CRP are non-specific for evaluation of UC solely (19). Theede *et al.* study indicated that FC levels are more sensitive and specific than ESR and CRP in assessment of UC severity (20). Moreover one study looking at the correlation between CRP or ESR and colonscopic activity in patients with UC revealed that there is only a very modest correlation between them and unlike FC, ESR and CRP could not be considered as an assay biomarker (21). 

In a meta-analysis, Yarlas *et al.* have studied the burden of UC on patients’ quality-of-life by synthesizing data from studies comparing scores from the SF-36 between UC patients and matched reference samples. They have been concluded that UC patients with active disease experience burdens on physical, emotional, and social functioning and well-being, and that normalization of these outcomes is observed in patients with inactive UC (22). So we have compared SF-36 between melatonin and placebo groups in UC patients for the first time. The result of our study indicated better scores in some of subdomain such as role-emotional, energy, and general health in melatonin compared with the placebo group significantly (*p* = 0.050, *p* = 0.002, and *p* = 0.037, respectively). A number of studies have been reported better quality of life by administration of melatonin in other diseases. For example, Russcher *et al.* have demonstrated that in hemodialysis patients, melatonin 3 mg/d could enhanced general mental health versus placebo (*p* = 0.05). Also emotional role activities and last year’s health change tended to improve with melatonin (difference 29.8% and difference 14.6% respectively) (23). Grima *et al.* have been evaluated effect of melatonin 2 mg/d versus placebo in sleep disturbances and quality of life in patients with traumatic brain injury. With respect to SF-36, melatonin improved self-reported vitality and mental health. Of course melatonin was not associated with important changes on the other six domains of the SF-36 (24).

In our study, only one patient has been excluded from the study because of possible severe nightmares related to the placebo. The patients reported no severe adverse drug reaction either by melatonin or placebo group in the present study, a consonant finding with previous studies which used the same dose of melatonin (3 mg/d) for the other purposes (25, 26). In Chojancki *et al.* study, some participants reported recurrent headaches felt, but probably not related to melatonin or placebo, and no serious adverse events were reported too (7). 

As Chojancki *et al.* trial have been continued up to 12 months, they could evaluate the effect of melatonin on some psychosomatic such as anxiety and intensity of depression in UC patients too. Although they have reported that melatonin 5 mg/d compared with placebo did not change anxiety or intensity of depression after 12 months. Our trial was limited to three months, so assessment of the above parameters was not possible. Moreover, it is possible that this dose (3 mg/d) is not strong enough for a positive response in all components of SF-36. In the future, we recommend doing trials by using higher doses of melatonin on clinical, biochemical, and quality of life in UC patients. 

**Table 1:** Baseline characteristics of the patients in control and intervention arms of the study

***P-*** **value**	**Control group (n = 15)**	**Intervention group (n = 15)**	**Variables**
0.726	8/7	7/8	Female/ Male gender (No.)
0.098	39.60 ± 10.98	33.66 ± 7.72	Age (year)
0.608	23.39 ± 3.43	24.04 ± 3.40	Body mass index
0. 860	8.46 ± 3.41	8.2 ± 4.70	Length of the disease(year)
0.601	468.31 ± 304.48	401.27 ± 311.12	Baseline Fecal calprotectin (mg/kg)
0.775	20.33 ± 16.23	22.27 ± 20.30	Baseline Erythrocyte sedimentation rate (mm/h)
0.624	7.60 ± 8.22	12.53 ± 23.65	Baseline C-reactive protein (mg/L)

**Table 2:** Summary of the health-related quality of life parameters overtime

***P*** **-value**	**control group (n = 15)**	**Intervention group ** **(n = 15)**	**Variables**
**Physical function**			
0.954	84 ± 17.24	84.33 ± 14	Base line
0.849	91 ± 15.27	90 ± 12.68	3 months
**Role-physical**			
0.906	60 ± 40.97	56.67 ± 34.68	Base line
0.967	75 ± 29.88	73.33 ± 25.82	3 months
**Role-emotional**			
0.858	33.34 ± 37.80	34.98 ± 36.76	Base line
0.050	51.09 ± 39.59	66.66 ± 35.64	3 months
	**Bodily pain**		
0.619	66.33 ± 21.27	70.33 ± 22.30	Base line
0.370	73.67 ± 20.61	80.67 ± 21.43	3 months
**Energy**			
0.063	44.67 ± 18.75	55.67 ± 11.47	Base line
0.002	47.67 ± 16.35	65.33 ± 11.72	3 months
**General health**			
0.893	54 ± 19.10	53 ± 21.03	Base line
0.037	50.67 ± 24.12	70.33 ± 25.18	3 months
**Health change**			
0.806	65 ± 37.55	63.33 ± 33.89	Base line
1.000	70 ± 34.33	70 ± 30.17	3 months
**Social function**			
0.669	65.33 ± 33.94	70 ± 24.46	Base line
0.47	67.50 ± 31.27	75 ± 25	3 months
**Mental health**			
0.388	57.07 ± 24.45	64.53 ± 22.16	Base line
0.161	58.93 ± 25.45	71.47 ± 22.11	3 months

**Table 3.** Biochemical markers at the end of study in two groups

***P*** **-value**	**control group (n = 15)**	**Intervention group ** **(n = 15)**	**Variables**
0.004	396.69 ± 251.95	119.25 ± 88.42	Fecal calprotectin (mg/kg)
0.697	14.86 ± 12.75	16.80 ± 14.58	Erythrocyte sedimentation rate (mm/h)
0.461	6.93 ± 11.78	11.86 ± 26.45	C-reactive protein (mg/L)

## Conclusion

The result of our study suggests that adjuvant melatonin therapy (3 mg/d) is relatively safe, and it may help in the improvement of clinical signs and symptoms, decrease of FC, and optimization of some components of quality of life (including role-emotional, energy, and general health) in mild to moderate UC patients.
